# Multi locus sequence typing of *Burkholderia pseudomallei* isolates from India unveils molecular diversity and confers regional association in Southeast Asia

**DOI:** 10.1371/journal.pntd.0006558

**Published:** 2018-06-27

**Authors:** Veeraraghavan Balaji, Susmitha Perumalla, Rajamani Perumal, Francis Yesurajan Inbanathan, Suresh Kumar Rajamani Sekar, Miracle Magdelene Paul, Rani Diana Sahni, John Antony Jude Prakash, Ramya Iyadurai

**Affiliations:** 1 Department of Clinical Microbiology, Christian Medical College, Vellore, Tamilnadu, India; 2 Department of Orthopaedics, Christian Medical College, Vellore, Tamilnadu, India; 3 Department of Medicine, Christian Medical College, Vellore, Tamilnadu, India; Charles Darwin University, AUSTRALIA

## Abstract

**Objectives:**

*Burkholderia pseudomallei*, the causative agent for melioidosis, has become a public health problem in India and across the world. Melioidosis can be difficult to diagnose because of the inconsistent clinical presentations of the disease. This study aims to determine the genetic diversity among the clinical isolates of *B*. *pseudomaelli* from India in order to establish a molecular epidemiology and elucidate the Southeast Asian association.

**Methods:**

Molecular typing using multi locus sequence typing was performed on thirty one archived *B*. *pseudomallei* clinical isolates, previously characterised from specimens obtained from patients admitted to the Christian Medical College & Hospital, Vellore from 2015 to 2016. Further investigations into the genetic heterogeneity and evolution at a regional and global level were performed using *insilico* tools.

**Results:**

Multi locus sequence typing (MLST) of the isolates from systemic and localized forms of melioidosis, including blood, pus, tissue, and urine specimens, revealed twenty isolates with novel sequence types and eleven with previously reported sequence types. High genetic diversity was observed using MLST with a strong association within the Southeast Asian region.

**Conclusions:**

Molecular typing of *B*. *pseudomallei* clinical isolates using MLST revealed high genetic diversity and provided a baseline molecular epidemiology of the disease in India with a strong Southeast Asian association of the strains. Future studies should focus on whole genome based Single-Nucleotide-Polymorphism (SNP) which has the advantage of a high discriminatory power, to further understand the novel sequence types reported in this study.

## Author summary

*Burkholderia pseudomallei*, a gram negative bacteria, is the causative agent for melioidosis. Annually, around 165,000 people suffer from melioidosis worldwide. *B*. *pseudomallei* is present in wet soil and stagnant water. It enters the human body via percutaneous inoculation, inhalation, aspiration, and occasionally ingestion. Clinical presentations of *B*. *pseudomallei* vary by geographical region. Melioidosis occurs predominantly in Southeast Asia, northern Australia, South Asia (including India), and China. Occasional cases occur in other countries around the world. Melioidosis has become a public health problem in India, due to the increasing numbers of people affected in various parts of the country. This study provides baseline data on the genetic diversity among *B*. *pseudomallei* isolates from different clinical samples (blood, pus, tissue and urine) of patients admitted to a tertiary care hospital using signature nucleotide sequences via multi locus sequence typing (MLST). Further, this study shows a relationship among *B*. *pseudomallei* previously reported in various Southeast Asian countries over the years from 1935 and 1947 with those seen in current clinical cases.

## Introduction

*Burkholderia pseudomallei*, the causative agent of the infectious disease melioidosis, is estimated to cause 165,000 cases of human melioidosis per year worldwide [1]. *B*. *pseudomallei*, an environmental saprophyte is commonly found in wet soil and stagnant water throughout endemic regions. The mode of infection is by inhalation, through cuts in the skin, and occasionally through ingestion [2]. The most severe clinical manifestation is melioidosis septic shock, which is often associated with pneumonia and bacterial dissemination to distant sites [3]. Melioidosis often affects individuals with one or more pre-existing conditions associated with an altered immune response,the most common being diabetes mellitus [4,5].

Melioidosis is endemic to Southeast Asia and Northern Australia, but still can be recognized in other countries worldwide [6]. Regional variations in clinical presentation of melioidosis are widely observed such as the predominance of pivotal swelling seen in Australia but not in Thailand. The contributing factors for this diversity are still unclear whether bacterial, host or environmental. There is no correlation between the clinical presentations and genotypes to date, even though environmental partitioning between Australian and Asian population of *B*. *pseudomallei* have been reported previously [7]. Melioidosis has become a public health problem in India, due to the steady rise in case detection rates from various parts of the country. Moreover, no consistency has been observed in the forms of melioidosis (clinical presentations) reported among the sporadic cases across the country in the last two decades. A recent report reveals genetic diversity among clinical isolates of *B*. *pseudomallei* from South India [8]. This study focuses on the clinical manifestations and genetic diversity of *B*. *pseudomallei* isolated from patients across India using the multi locus sequence typing (MLST) scheme for *B*. *pseudomallei* as described by PubMLST, with an attempt to establish the molecular epidemiology in Southeast Asian region.

## Methods

A total of 31 *B*. *pseudomallei* clinical isolates that were previously characterised from different clinical specimens (blood, pus, tissue and urine) obtained from patients admitted to the Christian Medical College & Hospital, Vellore from different parts of the country, during 2015 to 2016 were included in this study. The total genomic DNA was extracted using automated method (QIASymphony SP, QIAGEN, Germany). MLST was performed by PCR amplification of seven house-keeping genes (*ace*, *gltB*, *gmhD*, *lepA*, *lipA*, *narK*, *ndh*). The primer sets used for PCR amplification were obtained from *B*. *pseudomallei* MLST scheme as described in PubMLST (https://pubmlst.org/bpseudomallei/). Sequencing PCR using the same primer set was performed using the Big Dye Terminator (v3.1) cycle sequencing kit (Applied Biosystems, Thermo Fisher Scientific Company, Waltham, MA.) under the manufacturer’s protocol, purified and resolved on ABI 3500 Genetic Analyzer (Applied Biosystems, Thermo Fisher Scientific Company, Waltham, MA). The complete sequences of the seven loci of the house keeping genes were assigned allelic number and defined a sequence type based on the allelic profile match on the PubMLST database. New allele numbers and STs were assigned to sequences not reported previously by submission to the database. Genetic relatedness of the isolates in comparison to the global isolates was analysed using the goeBURST algorithm of the PHYLOVIZ open source software to establish a clonal association. The nucleotide diversity was calculated using the allelic sequences on the DNA sequence polymorphism software (v6.10.01). SplitsTree4 (version 4.14.6) was used to derive a comparative phylogenetic relationship among the study isolates and isolates previously reported from India (http://www.ub.edu/dnasp/).

### Ethics statement

The *B*. *pseudomallei* are from clinical specimens (blood, pus, tissue and urine) from patients admitted to the Christian Medical College and Hospital, Vellore. This study was approved with ethical clearance to use the clinical isolates by the Institutional Review Board with IRB MIN 16044. The clinical samples are anonymized.

## Results

Within the thirty one clinical isolates obtained during 2015 to 2016, Systemic forms of melioidosis (blood) contributed to 51.6% (n = 16) and localized to 45% (n = 14) with one urinary tract infection (3.2%). In the case of the localised infections, the number of isolates from pus and the urinary tract infection were 29% (n = 9), tissue 16% (n = 5) respectively. Infected patients were from the states of Tamilnadu (n = 11; 35.5%), West Bengal (n = 5; 16.1%), Andhra Pradesh (n = 6; 19.5%), Jharkhand (n = 5; 16.1%), Kerala (n = 1; 3.2%) and Tripura (n = 1; 32%). Twenty isolates had distinct allelic profiles from the existing database and were assigned new sequence types (ST) (13 new STs, ST1630-ST1642). Eleven isolates in this study were found to be previously reported STs (ST51, ST1364, ST1099, ST300, ST1552, ST375, ST56, ST71, ST228 and ST99).

**Fig 1:**
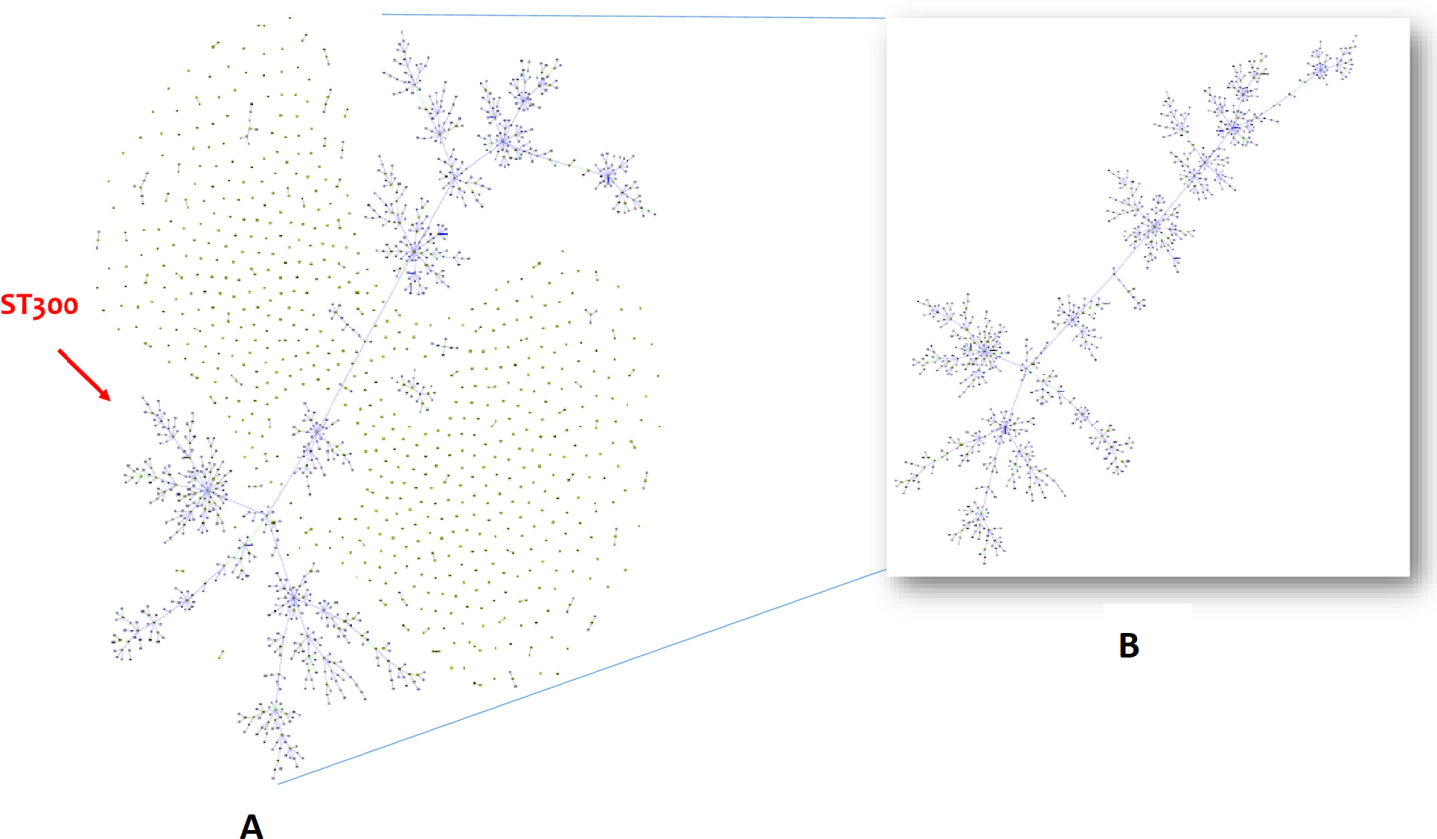
Populations structure analysis. **(A)** goeBURST analysis of 1643 STs present in the PubMLST database. Each dot represents the single ST. Groups are formed by linking the STs that are double locus variants (DLV) and called as clonal complex (CC). The largest clonal complex 300 has the other STs leaving 1634, 1632, 1636, 1639 as singletons (**B**) Snapshot of the clonal complex 300.

The genetic diversity among the identified STs was low as four isolates had ST1630 (12.9%), three had ST1639 (9.7%), two had ST51, ST1637, 1641 (6.5%) and the remaining 19 isolates having different STs. Isolate identifiers with geographical location, type of specimen and the corresponding STs are represented in Table 1. Interestingly in two patients with both systemic and localized infections (VBBP002 –Systemic–ST1630 and VBBP006 –localized—ST1631; VBBP011—Systemic–ST51 and VBBP023—localized–ST375), different STs were observed with respect to the site of isolation—they are single loci variants. goeBURSTanalysis clustered 18 of the study STs into a single major clonal complex of founder ST300. Four STs (1634, 1632, 1636, and 1639) were found to be singletons and are outliers (Fig 1).

**Table 1:** Details of the study isolates identifiers and corresponding year of isolation, type of infection, geographical location, and the sequence type information.

Isolate	Year	Type on infection	Type of Specimen	Patient Location	Sequence type
VBBP001	2016	Localized	Pus	West Bengal	1639 *
VBBP002	2016	Systemic	Blood	Tamilnadu	1630 *
VBBP003	2016	Systemic	Blood	Tamilnadu	1635 *
VBBP004	2016	Systemic	Blood	Jharkhand	1632*
VBBP005	2016	Localized	Urine	West Bengal	1633*
VBBP006	2016	Localized	Pus	Tamilnadu	1631*
VBBP007	2015	Systemic	Blood	Andhra Pradesh	1636*
VBBP008	2015	Systemic	Blood	Andhra Pradesh	1634*
VBBP009	2015	Systemic	Blood	Jharkhand	1637*
VBBP010	2015	Systemic	Blood	Jharkhand	1638*
VBBP011	2016	Systemic	Blood	Tamilnadu	51
VBBP013	2016	Systemic	Blood	Tamilnadu	51
VBBP014	2016	Localized	Pus	Andhra Pradesh	1630*
VBBP015	2015	Localized	Tissue	Kerala	1364
VBBP016	2016	Systemic	Blood	Jharkhand	1099
VBBP017	2016	Systemic	Blood	Tamilnadu	1630*
VBBP018	2016	Systemic	Blood	Tamilnadu	1639*
VBBP019	2016	Localized	Pus	West Bengal	1639*
VBBP020	2016	Localized	Pus	West Bengal	300
VBBP021	2016	Localized	Tissue	Tamilnadu	1630*
VBBP022	2016	Localized	Tissue	Tamilnadu	1552
VBBP023	2016	Localized	Tissue	Tamilnadu	375
VBBP024	2015	Localized	Pus	Jharkhand	1637*
VBBP025	2015	Localized	Pus	West Bengal	56
VBBP026	2015	Localized	Pus	Tripura	71
VBBP027	2015	Systemic	Blood	West Bengal	228
VBBP028	2015	Localized	Tissue	Bangladesh	99
VBBP029	2016	Systemic	Blood	Andhra Pradesh	1640 *
VBBP030	2016	Systemic	Blood	Andhra Pradesh	1641*
VBBP031	2016	Systemic	Blood	Tamilnadu	1642*
VBBP032	2016	Localized	Pus	Andhra Pradesh	1641*

* are the novel sequence types identified in this study.

**Fig 2:**
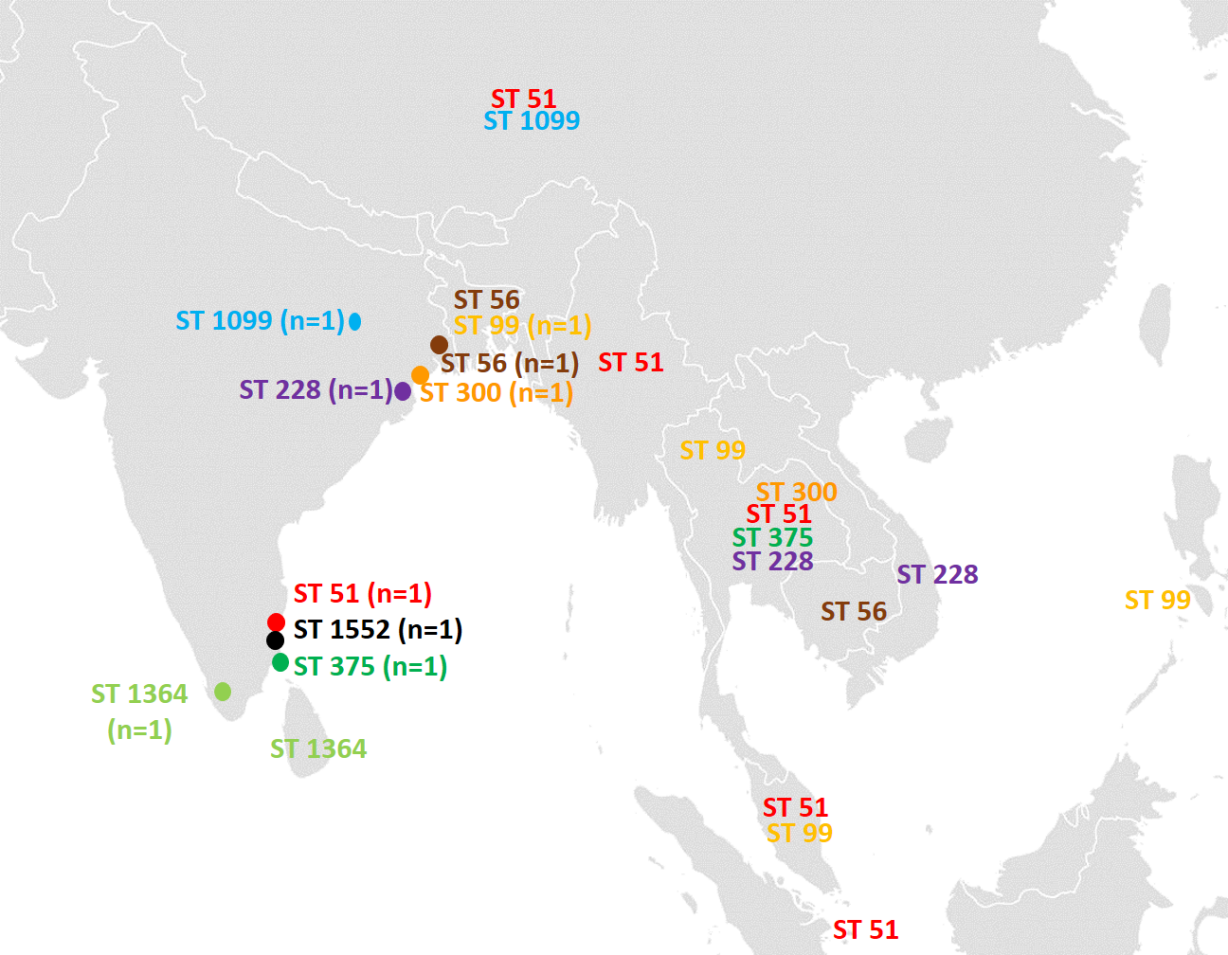
Distribution and association of the study ST’s with the ST’s from Southeast Asian region retrieved from the PubMLST database (10). Light Green–ST1364 (Kerala & Sri Lanka); Dark Green ST375 (Tamilnadu & Thailand); Black—ST1552 (Tamilnadu and Pondicherry); Red—ST51 (Tamilnadu, Singapore, China, Thailand, Malaysia and Burma); Purple—ST228 (West Bengal, Thailand &Vietnam); Blue–ST1099 (Jharkhand & China); Brown–ST56 (West Bengal Bangladesh, Cambodia & Vietnam); Orange—ST300 (West Bengal and Thailand); Yellow–ST99 (Bangladesh, Philippines, Thailand and Malaysia). The figure was recreated using open source-https://commons.wikimedia.org/wiki/Atlas_of_the_world.

Thirty five percent (n = 11) of the identified STs in this study have been previously reported and were found to be associated with Singapore (ST51), China (ST51, ST1099), Thailand (ST51, ST99, ST375, ST228, ST300), Malaysia (ST51, ST99), Burma (ST51), Bangladesh (ST56), Cambodia (ST56), Vietnam (ST56), Philippines (ST99) and Sri Lanka (ST1364) of Southeast Asia (Fig 2) [9, 10].

Except for the isolates with the ST51 (6.5%) and ST 56 (3%), no association was found between the epidemiological year and the prevalence of the isolates. ST51 and ST 56 were first observed in 1935 and 1947 and are still seen in clinical cases (Table 2).

**Table 2:** Epidemiological year of isolation of the STs identified in the study isolates from different South Asian countries.

Sequence Type	Country	Continent	Year of isolation
ST51	Singapore, China, Thailand, Malaysia, Burma	Asia	1935–2015
ST56	Bangladesh, Cambodia, Vietnam	Asia	1947, 1983, 1984, 1999, 2007, 2008, 2013, 2015
ST71	Unknown		1999, 2000
ST99	Philippines, Thailand, Malaysia	Asia	1964, 1966, 1969, 2006, 2007, 2015
ST375	Thailand	Asia	1966
ST228	Thailand, Vietnam	Asia	1965, 2001, 2010, 2016
ST1552	Pondicherry, India	Asia	Unknown
ST300	Thailand, India (Orissa)	Asia	1965, 2000, 2014, 2015
ST1364	Sri Lanka	Asia	2015
ST1099	China	Asia	2011

**Fig 3:**
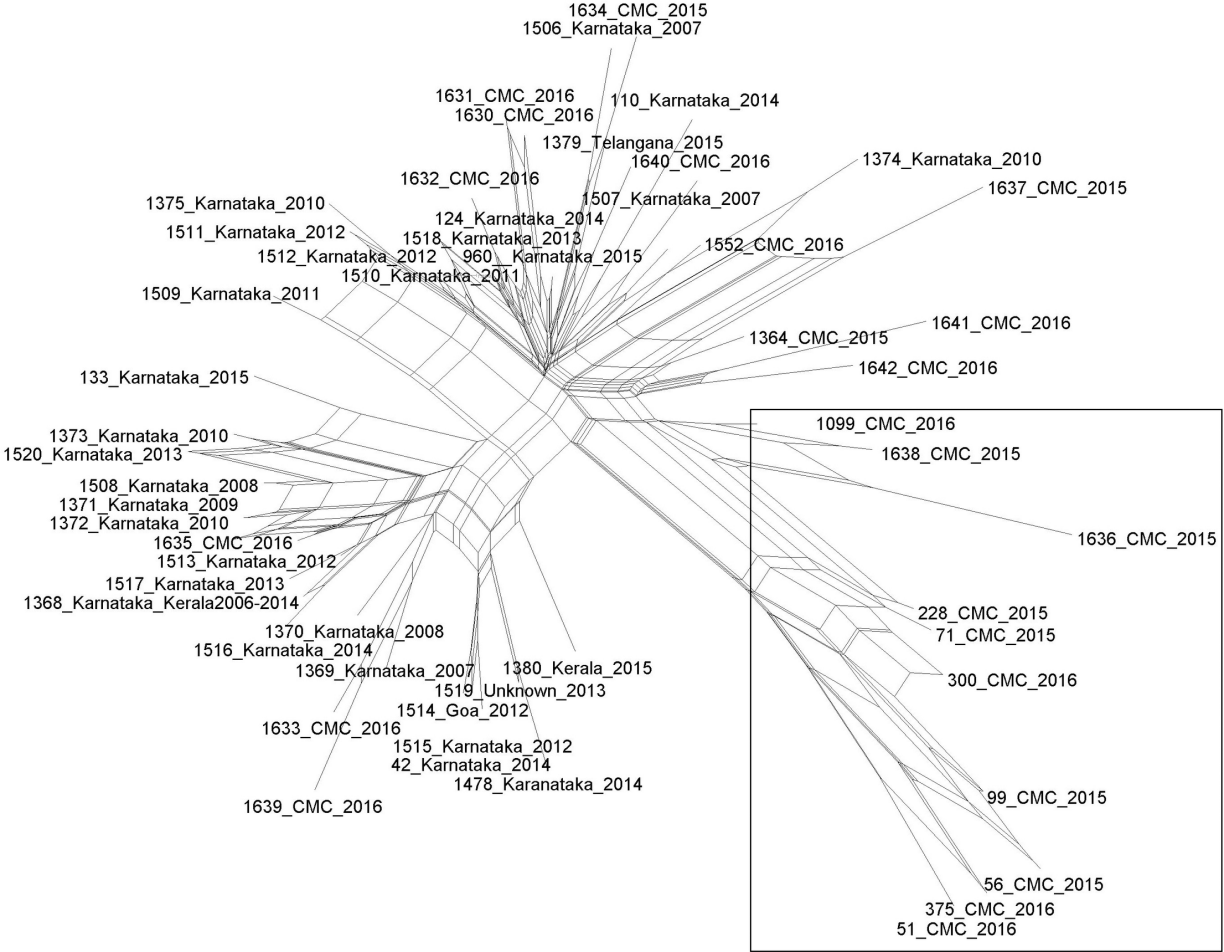
Splits tree of the concatenated allelic sequences of study isolates and previously reported South Indian isolates. STs within the square represents the STs associated to South East Asia reported in the study. It is an unrooted tree with bootstrap value of 1000.

Nucleotide diversity among the study isolates as calculated by DNA SP6 was 0.00212 and within the Indian isolates was 0.00182 (Table 3). Splits tree analysis depicts 80% of the isolates associated with Southeast Asia into one group wherein the rest of the study isolates are grouped differently (Fig 3).

**Table 3:** Comparison between the nucleotide diversity of MLST housekeeping genes of the study isolates.

Gene	Size in bp	Alleles	No of Polymorphic sites	Nucleotide Diversity
*ace*	519	3	2	0.00142
*gltB*	522	5	5	0.00224
*gmhD*	468	7	5	0.00306
*lepA*	486	4	3	0.00190
*lipA*	402	3	3	0.00155
*narK*	561	6	7	0.00318
*ndh*	443	2	1	0.00118
Concatenated Sequences	3401	23 STs	26	0.00212
Concatenated Indian Isolates	3401	54 STs	28	0.00182

## Discussion

The burden of *B*. *pseudomallei* in India and across the world is of a great concern due to its wide distribution in the community as an environmental etiological agent. Molecular typing by MLST method serves as a powerful epidemiological tool to determine the source of infection (local epidemiology) and understand the diversity and evolution of the pathogen population. Though this study involved a small number of isolates (n = 31), the identified STs in this study provide information on regional and country wide sequence diversity.

The different types of melioidosis in the Southeast Asian region include bacteraemia, skin/soft tissue infections, localized abscesses (splenic, prostatic, liver, prostatic, parotid), pneumonia and genitourinary tract infections [11]. There was no association found between the different types of melioidosis and the sequence types among the study isolates, which showed high diversity.

Thirty five percent (n = 11) of the study isolates are confined to STs of South East Asia conferring a regional association and the remaining are novel STs. Though there is high diversity among *B*. *pseudomallei* across India and South East Asia, this study provides insights into the regional STs corresponding to both systemic and localized infections being consistent over a long period of time. The correlation between a few of the identified STs (51, 56) and the epidemiological years denote persistent strains causing infection across the continent.

The variation among isolates (VBBP002:VBBP006 and VBBP011:VBBP023) in both systemic and localized infections from the same patient with a single loci variation shows possible evolution over a short period of time; however genome wide studies are needed to provide valid information. Nucleotide diversity and splits network analysis within the house keeping genes shows the least differences for the 6 housekeeping genes, reducing the possibilities of recombination events but more of single nucleotide polymorphisms. The nucleotide diversity of the *gmhD* gene was found to be (0.00306) and has the maximum number of allelic profiles within the study population. The existence of the parallelogram type of split tree network of all the south Indian isolates signifies the possibility of recombination events, but the Southeast Asian study isolates did not show a typical parallelogram and lie on the same group conferring the absence of recombination events [12].

Though Multi locus sequence typing (MLST) is one of the most commonly used Single-Nucleotide-Polymorphism (SNP) based phylogeny with the use of seven housekeeping genes, it represents only 0.05% (3401 nucleotides) of the total bacterial genome of 7.4 million nucleotides, with less discriminatory potential being the main limitation in a closely related group or within the sequence type. However, whole genome based SNP typing provides phylogeny with high discriminatory power, which could further type the isolates belonging to same sequence types and/or clonal group. This was substantiated by the studies done by Price et al., 2015, to show the differences between same sequence types but polyclonal by Whole Genome Sequencing in a patient with chronic melioidosis across years unveiling the genome plasticity [13], but the study did not indicate the non synonymous nucleotide polymorphisms. Additionally, Chapple et al 2016 describe the differences in *B*. *pseudomallei* by Whole Genome Sequences within the same sequence types being persistent across years and different regions, but the mutations not correlating to the environment factors [14]. This evidence gives a glimpse of the high evolution in *B*. *pseudomallei*, with conserving the core genome having strong ancestral relationships as derived in this study.

Prospective studies based on whole genome phylogeny would provide higher resolution over the genome plasticity of *B*. *pseudomallei* in India and the regional association through the conserved regions on this pathogen. Continent wide large scale genomic studies would enable us to establish a regional association of the strains [15]. To conclude, future studies must focus on whole genome based SNP typing in order to understand the phylogeny and evolution of this bacterium.
